# COVID-19: a novel menace for the practice of nephrology and how to manage it with minor devastation?

**DOI:** 10.1080/0886022X.2020.1797791

**Published:** 2020-07-27

**Authors:** Sena Ulu, Ozkan Gungor, Ebru Gok Oguz, Nuri Baris Hasbal, Didem Turgut, Mustafa Arici

**Affiliations:** a Department of Nephrology, Afyonkarahisar Health Sciences University School of Medicine, Afyonkarahisar, Turkey; b Department of Nephrology, Kahramanmaras Sutcu Imam University School of Medicine, Kahramanmaras, Turkey; c Department of Nephrology, Diskapi Yildirim Beyazit Training and Research Hospital, University of Health Sciences, Ankara, Turkey; d Department of Nephrology, Hakkari State Hospital, Merkez, Hakkari, Turkey; e Department of Nephrology, Baskent University School of Medicine, Ankara, Turkey; f Department of Nephrology, Hacettepe University School of Medicine, Ankara, Turkey

**Keywords:** COVID-19, hypertension, acute kidney injury, hemodialysis, renal transplantation, glomerulonephritis

## Abstract

Coronavirus disease 19 (COVID-19) became a nightmare for the world since December 2019. Although the disease affects people at any age; elderly patients and those with comorbidities were more affected. Everyday nephrologists see patients with hypertension, chronic kidney disease, maintenance dialysis treatment or kidney transplant who are also high-risk groups for the COVID-19. Beyond that, COVID-19 or severe acute respiratory syndrome (SARS) due to infection may directly affect kidney functions. This broad spectrum of COVID-19 influence on kidney patients and kidney functions obviously necessitate an up to date management policy for nephrological care. This review overviews and purifies recently published literature in a question to answer format for the practicing nephrologists that will often encounter COVID-19 and kidney related cases during the pandemic times.

## Introduction

The world is in a great struggle with a disease named Coronavirus Disease 19 (COVID-19) for about six months. A newly identified member of a known coronavirus family 'Severe Acute Respiratory Syndrome Coronavirus-2′ (SARS-CoV-2) is the responsible virus [1,2]. SARS-CoV-2 seems to be more contagious compared to other members of the family [3]. The outbreak emerged first in Wuhan, China at the end of December 2019. Infection has been reported in all ages, but recent epidemiological studies have clearly showed that elderly people with chronic diseases are likely to have a severe course [4]. Early studies from China showed that hypertension (63%), diabetes (21%), and cardiovascular diseases (14%) were common accompanying diseases, especially in patients who did not recover [5].

Nephrologists are in the second line of the front, immediately after infectious disease specialists, intensivists and pulmonologists against COVID-19. Many of our patients are under risk for COVID-19, and many patients with COVID-19 ended up with kidney problems. Infected patients usually experienced moderate to severe disease with increased rates of hospitalization and mortality. Many practices except nephrology have the chance to slow down their usual daily burden. However, at least dialysis care should continue in every situation whether there is a disaster or a COVID-19 pandemic. Although some patients in some countries have experienced difficulties in continuing their dialysis schedules, there is also a significant physical/psychological and economic burden for the dialysis practice. Severe acute kidney injury is also a mortal condition where nephrological intervention is mandatory.

The widespread threat of COVID-19 caused an enormous increase in relevant publications/recommendations. It became almost impossible for the practicing nephrologists to follow them while fighting for the COVID-19 and kidney related problems. This review thus overviews and purifies recently published literature in a question to answer format for the practicing nephrologists.

## COVID-19 and acute kidney injury

### Question 1: Does SARS-CoV-2 have direct effect on kidney cells? If yes, what is the mechanism?

Answer: The first step in Coronavirus infection is entry of the virus into the cell by binding host cell receptor. SARS-CoV-2 has a similar protein structure with SARS-CoV and attaches to the cell surface by angiotensin-converting enzyme 2 (ACE 2) receptor [6]. The infection mainly affects lower respiratory tract; but may also be presented with multiorgan involvement. Single-cell RNA sequencing analysis showed that ACE2 receptors were located on type 2 alveolar cells, myocardial cells, kidney proximal tubule cells, esophageal epithelial cells, and bladder uroepithelial cells [7]. Lungs, heart, esophagus, kidney, bladder or ileum become the target organs for COVID-19. ACE2 receptor is found mainly on the proximal tubular apical brush border and less frequently on podocytes. It is absent on the endothelial cells of the kidney [8]. Su et al. showed that tubular and glomerular visceral epithelial cells are the primary target cells for SARS-CoV-2 rather than the endothelial cells in the postmortem biopsies [9]. This makes tubular and glomerular visceral epithelial cells prone to the cytopathic effects of the virus in case of viremia. However, the data is still controversial about the target cells or tissues. SARS-CoV-2 RNA was also detected in the urine of patients with acute kidney injury (AKI) [10]. Direct toxic effects of the virus on kidneys are not apparent yet, but in the case of viremia, they would be a reservoir causing contamination during the infection course.

### Question 2: Does COVID-19 cause acute kidney injury (AKI)? What is the incidence?

Answer: Complications of disease or disease itself may result in AKI. It is challenging to say the real incidence in the amid of available data. Patients with severe infection (who need ICU follow-up or intubation) had a high incidence of AKI (18–37.5%), while it was low (0.5 − 15%) in mild-to-moderate infection [10–12]. The potential causes of AKI are summarized in Table 1.

**Table 1.** Potential causes of AKI in COVID-19 patients.

Renal	Non-renal
Direct renal parenchymal infection Acute tubular injury Podocyte injury Fibrin thrombus or fibrinoid necrosis	Rhabdomyolysis-associated pigment nephropathy Cytokine release syndrome Sepsis-associated multi-organ failure Nephrotoxicity related to diagnostic and therapeutic interventions Cardiorenal syndrome—heart-kidney crosstalk and lung-kidney axis

### Question 3: What are the lab results in COVID-19 related AKI?

Answer: In a prospective study involving 701 patients, increased serum creatinine and blood urea nitrogen (BUN) were observed in 14.4 and 13.1% of patients, respectively [13]. Also, proteinuria and hematuria were observed 43.9 and 26.7% of the patients, respectively in the same study. There was no data about pyuria. The patients with elevated creatinine levels on admission were older, dominantly male, and had more severe COVID-19 infection. They had higher leukocyte, lower lymphocyte and platelet levels, and higher D-Dimer levels with serious coagulation problems. Aspartate aminotransferase, lactate dehydrogenase, and procalcitonin were also higher in this patient group. Patients with normal baseline creatinine levels had a lower incidence of AKI.

### Question 4: What do we know about early renal injury in patients with COVID-19?

Answer: In a study from Wuhan, 83 patients who had urinalysis at admission of total 178 patients with COVID-19 were evaluated. 34.9% of patients were with proteinuria, 31.3% of patients were with hematuria [14]. Patients with proteinuria or hematuria had a worse disease course with liver injury, higher inflammatory markers and serious coagulation problems. It was argued that kidney involvement could be an early sign of severe infection. In a case series as a preprint, 12 patients with COVID-19 were reported [15]. There was not a significant increase in serum creatinine and BUN. eGFR declined in 66.7% of patients, and 24-h creatinine clearance dropped in 41.7%. Urinary microalbumin/creatinine ratio increased in 41.7% of patients. Early renal damage markers, such as β1 − microglobulin, urinary immunoglobulin G, and urinary transferrin levels were also increased. All these findings may reveal an initial renal injury in the course of COVID-19. It is, however, difficult to speculate whether it is related to infection itself.

### Question 5: What are the kidney biopsy findings in COVID-19 related AKI?

Answer: In a postmortem analysis of 26 COVID-19 patients (only 9 patients had increased serum creatinine and/or new-onset proteinuria); severe acute tubular injury, endothelial cell damage, and occlusion of microvascular lumens with erythrocytes were major findings [9]. Glomerular and vascular changes showing diabetic and hypertensive processes were also observed. Electron microscopy demonstrated spherical virus particles, which belong to the SARS-CoV-2 in the proximal tubular epithelium. Direct invasion of virus particles to the podocytes and podocyte injury like foot process effacement were determined. The paper had not specified whether the patients with viral invasion had renal function problems. Erythrocyte accumulation-fragments, fibrin thrombus, and fibrinoid necrosis were clearly seen in glomerular and peritubular capillaries. Pigmented casts were observed in patients with high serum creatinine kinase. Nonspecific IgM and C3 trapping were observed in the immunofluorescence examination of 6 patients. In terms of differential diagnosis, interstitial hemorrhage, which is a characteristic finding of Hantavirus infection or crescentic glomerular injury, as seen in pulmonary-renal syndromes, were not observed. In a case report of a COVID-19 infected patient with AKI, kidney biopsy showed severe collapsing focal segmental glomerulosclerosis [16].

### Question 6: Does AKI in COVID-19 has an increased impact on morbidity and mortality?

Answer: In a prospective cohort study of 701 patients with COVID-19, while serum albumin levels were significantly low in patients who died from COVID-19; blood urea nitrogen, serum creatinine, serum potassium, creatinine kinase, and lactate dehydrogenase levels were found to be significantly high [13]. Proteinuria, hematuria, increased BUN-creatinine levels, and AKI were independent risk factors for in-hospital deaths, even after correcting for age, gender, disease severity, comorbid diseases, and lymphocyte count. In a meta-analysis, Ali H et al. demonstrated that AKI is associated with higher mortality in patients with COVID-19 [17]. Considering the effects of kidney findings on morbidity and mortality of the disease, it is essential to follow-up renal functions meticulously in all COVID-19 patients.

### Question 7: How should AKI is managed in the course of COVID-19 infection?

Answer: Underlying prerenal, renal, or postrenal causes of AKI should be evaluated like routine AKI management. Physicians should be cautious about contrast exposure. Medications should be reviewed daily for their potential nephrotoxicity. Keeping patients in euvolemic state is essential to protect them from hypovolemia in terms of sepsis and from hypervolemia in terms of ARDS. Renal replacement therapy (RRT) indications for COVID-19 related AKI is not different than any other AKI. Early start of RRTs may increase contamination risk for health-care workers and increase the burden for nephrology practice.

### Question 8: Which dialysis modality may be chosen for COVID-19 patients with AKI?

Answer: There is no clear data to answer this question. Critical course of infection and rate of pandemic make health systems too vulnerable to function. Both health-care workers and dialysis equipment may be insufficient if critically ill patients’ numbers explode. In such cases, PD should be an RRT option for AKI. PD is a good way in hemodynamically unstable patients with less equipment necessity. Early mechanical complications related to PD catheterization would be ignored under optimal techniques [18]. It is controversial whether continuous replacement therapies (CRRTs) suggest a difference in outcomes for critically ill patients. Patients with AKI requiring renal replacement therapy are those with severe COVID-19 infection. CRRTs may be a strong alternative to intermittent hemodialysis (IHD) in patients with poor hemodynamic status or who needs inotropic agents or mechanical ventilation. Burgner et al. reported that CRRT could be performed by adding a hemofilter to the extracorporeal membrane oxygenation (ECMO) circuit [19]. In patients who end up on ECMO and need CRRT, continuous arteriovenous hemodiafiltration may be an option by this way. In this setting, ultrafiltration may be limited, which is critical for sepsis management. It is thought that the inflammatory response can be reduced with various cartidges containing highly biocompatible sorbents and microporous resins such as Jafron (Biomedical Co., China) and Cytosorb (CytoSorbents Corporation, NJ, USA) using in accordance with CRRTs. In case of cytokine release syndrome, which is common during severe infection, those cytokines with the highest concentration will be removed in higher amount by these cartidges and this phenomenon is called ‘peak concentration hypothesis’ [20]. Also, serum inflammatory cytokines (especially IL-6) removal in critically ill patients with high cutoff membranes is higher than the other membranes, but albumin loss should be kept in mind [21]. The cost of these membranes and absence of evidence about benefit in COVID-19 patients should be kept in mind.

### Question 9: Does antiviral drugs used in covid-19 treatment have nephrotoxic effects? Is dose adjustment necessary in case of AKI?

Answer: Renal pharmacokinetic properties of azithromycin, favipravir, hydroxychloroquine, lopinavir/ritonavir, remdesevir and tocilizumab were summarized in Table 2 [1].

**Table 2.** Antiviral drugs used in COVID-19.

Drug	Renal dose adjustment	Renal side effect	Additional feature
Azithromycin	Careful use if GFR <10 ml/min	Rarely AKI, interstitial nephritis	HD: No dose adjustment or supplemental dose necessary PD: No dose adjustment or supplemental dose necessary CRRT: No dose adjustment or supplemental dose necessary
Favipravir	No valid data	No valid data	Renal clearance No valid data for HD, PD or CRRT
Hydroxychloroquine	None	Risk of renal insufficiency in chronic use	Cannot be removed by dialysis HD: No dose adjustment (expert opinion) PD: No dose adjustment (expert opinion) CRRT: No dose adjustment (expert opinion)
Lopinavir/Ritonavir	No valid data	None	Dose adjustment is not necessary in HD patients Avoid once-daily dosing in HD patients No recommendation for PD and CRRT
Remdesevir	Do not use GFR <30 ml/min	No valid data	HD: Do not use PD: Do not use CRRT: Do not use
Tocilizumab	None	Nephrolithiasis	No valid data for HD, PD or CRRT

## Covid-19 and hypertension

### Question 1: Are patients with hypertension under increased risk for COVID-19? Is there any epidemiological relation?

Answer: Early published data about COVID-19 shows that hypertension (HT) was the commonest comorbidity in patients with severe infection. This may be due to increased number of elderly patients who are at risk of COVID-19 and its severe forms. Retrospective reports showed that up to 40% of patients had HT and they had worse disease courses [22]. In an Italian report, median age of the patients who died was 80.5, while the median age of all COVID-19 patients was 63 [23]. In the same report, it has been stated that HT frequency increases to 76% in patients who died. In a study with 4103 patients reported from USA; age, obesity, heart failure, low oxygen saturation, high D-dimer, ferritin, and CRP, but not HT, were independent risk factors for COVID-19 hospitalization and severe infection [24]. In a meta-analysis of Matsushita et al., 51,845 patients with COVID-19 were evaluated. Nine thousand sixty-six of patients were critically ill in this report. Although studies in the analysis were not in high quality; older age, male sex, HT, diabetes, and cardiovascular diseases were risk factors for severe COVID-19 [25]. Current data about COVID-19 and hypertension is mostly unadjusted for age. Hypertension is exceedingly frequent in the elderly, and older people appear to be at a particular risk of COVID-19 and its severe course. With the available information, a predisposition to COVID-19 or a worse disease course in HT is not likely.

### Question 2: What is the relationship between renin-angiotensin-aldosterone system (RAAS) blockers and COVID-19? Does antihypertensive medication type affect morbidity and mortality in the COVID-19?

Answer: The role of ACE2 (angiotensin-converting enzyme 2) receptors was proposed as a possible hypothesis for explaining the potential relationship between RAAS blockers and COVID-19. In 2003, Li et al. discovered that the spike protein of SARS-CoV uses the encoded protein of ACE2 as a functional receptor to enter the cell [26]. SARS-CoV-2 also uses this receptor for cell entry. It was hypothesized that high affinity of SARS-CoV-2 to ACE2 receptor along with the presence of ACE2 receptors in type II alveolar cells and macrophages in the lungs increases the infectivity of the virus. ACE2 is also expressed in small intestine, testes, heart, kidney. ACE2 acts as a counter-regulatory component of the renin-angiotensin-aldosterone system by catalyzing angiotensin 2 to vasodilator angiotensin 1–7. Unlike angiotensin 2, angiotensin 1–7 shows some organ protective effects by modestly lowering blood pressure, increasing renal sodium and water excretion and modulating inflammation [27].

There is a controversy about the effect of RAS blockers and their effect on ACE2 expression. It was hypothesized that ACE inhibitors or AT1 receptor blockers (ARBs) increase ACE2 levels, thus increase viral entry to the cells. There is experimental evidence that ACE inhibitors or ARBs may increase ACE2 levels in heart and kidney tissue in animals; but this has not been shown consistently in humans [28]. There is however a counterhypothesis in which RAAS blocker use may be beneficial in alleviating acute lung injury by decreasing angiotensin 2. This controversy and social media induced news cause great hesitancy among patients whether to continue using RAAS blockers.

Recent studies have almost abolished the hypothesis that RAAS blockers may have detrimental effect on the course of COVID-19. Reynolds et al. showed no relation between COVID-19 test positivity or disease severity concerning any antihypertensive drug type (including ACE inhibitors, ARBs, beta-blockers, calcium-channel blockers or thiazide diuretics) in a group of 12.594 patients [29]. Mancia et al. analyzed 6272 COVID-19 patients with healthy controls. They reported that ACEIs and ARB use were more frequent among COVID-19 patients, but there was no relation with the infection risk [30]. Mehra et al. also showed that there is no increased risk of in-hospital death with either use of ACE inhibitors or ARBs in a group of 8910 patients [31]. Regarding this data, there is no need to stop RAAS blockers in healthy HT patients. Patients should not be deprived of the long-term favorable mortality outcomes of RAS blockers. In patients with COVID-19, RAS blocker use may be beneficial (which is currently tested in some clinical trials) should only be discontinued with severe, critical illness and acute kidney injury. All data is unclear with low quality, but it may be recommended that; patients under treatment have to continue their current drug medications, considering the interaction with the antiviral drugs used for COVID-19.

### Question 3: Are there any drug interactions between antihypertensive drugs and antivirals used in the COVID-19?

Answer: There is limited data about the pharmacokinetics of antivirals (atazanavir, lopinavir/ritonavir, remdesivir, favipiravir, chloroquine, hydroxychloroquine, nitazoxanide, ribavarin, tocilizumab) used in the COVID-19. Concerns may be the followings [32]:

- ACEIs: Benazepril increases the level of atazanavir, and antiviral drug level monitoring is recommended. Fosinopril may increase the level of lopinavir/ritonavir, but blood level monitoring is not necessary.
- ARBs: Valsartan increases the level of atazanavir and lopinavir/ritonavir and should be used carefully. Irbesartan and losartan reduce the level of lopinavir/ritonavir slightly.
- Diuretics: Indapamide increases the blood level of atazanavir and lopinavir/ritonavir; close drug level monitoring is required for concomitant use.
- Calcium channel blockers: Concomitant use of lercanidipine with atazanavir or lopinavir/ritonavir is contraindicated. Other calcium channel blockers also interact with atazanavir, lopinavir/ritonavir, level monitoring is recommended.

## Covid-19 and dialysis

### Question 1: Are dialysis patients at increased risk for the COVID-19?

Answer: End-stage renal disease (ESRD) patients, especially hemodialysis (HD) patients, are at increased risk for COVID-19 and its severe course. These patients are usually middle-aged or elderly and have many comorbid conditions such as diabetes, hypertension, and cardiovascular diseases [33]. Predisposing factors in ESRD patients are mainly uremia related immune system weakening. Uremia itself alters both innate and adaptive immunity. Neutrophil and monocyte dysfunction, impaired T cell maturation, and decreased humoral response contribute to immunosuppression. HD patients need regular travels to dialysis units and patient clustering during the sessions increase the risk of infection spread among patients and staff. Home hemodialysis or peritoneal dialysis (PD) patients have more chance to maintain social distancing. Besides these features of dialysis patients, Vicenzi et al. demonstrated that heparin, a polyanion, reduced plaque formation in SARS-CoV infected Vero (monkey kidney epithelial cell) cell-line by 50% [34]. They emphasized that heparin may cause a decrease in viral attachment and entry of a cell in the light of previous studies about HIV and HSV. Therefore, patients undergoing hemodialysis who used heparin three times a week as an anti-coagulation may be affected less than the others.

### Question 2: Are there any data about infected dialysis patients since the beginning of the COVID-19 pandemic?

Answer: There are not any large-scale reports about HD patients yet. Fu et al. reported a 75-year-old patient with moderate coronavirus infection who was on HD for four years [35]. The patient took oseltamivir and umifenovir as antiviral treatment and successfully treated without any severe complication. In a case report, Ferrey et al. described a hemodialysis patient with COVID-19 infection who had gastrointestinal symptoms rather than respiratory tract ones [36]. Wang et al. presented data of five patients with COVID-19 pneumonia, located in the center of Wuhan, in which 201 chronic HD patients were located [37]. After the first COVID-19 case, the researchers screened all their HD patients with a chest computerized tomography (CT) and, if abnormal, a Polymerase Chain Reaction (PCR) testing for SARS-CoV-2 was done. They found 2.5% prevalence at that time. All patients had diarrhea rather than the classical symptoms of COVID-19, which points that HD patients may have an atypical presentation [37].

### Question 3: What are the recommendations for HD patients during the COVID-19 pandemic?

Answer: Education about preventive strategies for COVID-19 is fundamental. Hemodialysis patients should pay attention to social distance and hygiene rules. Patients should be aware of signs/symptoms of infection and have plans if they or family members are infected. In case of disease, patients should be transported to the dialysis unit with their private vehicle because of the contamination risk of other dialysis patients [33].

### Question 4. Which measures should be taken in outpatient dialysis units during the COVID-19 pandemic?

Answer: The following measures are critical in dialysis facilities;

- Education should be at the forefront of the steps to be taken. Nurses, technicians, dieticians, cleaning staff, and transport service drivers should be educated about this contagious disease. Signs and symptoms of the disease, transmission protection ways, use of personal protective equipment and what to do after contact with an infected person should be covered in interim policies. These policies should be repeated from time to time.
- HD patients should be screened for possible symptoms before they came to the center. Patients with fever or any upper respiratory tract symptom should be directed to the hospital before they arrived. To measure body temperature before the transport service may protect other patients from contamination during transport to the dialysis unit [33].
- During the transport, patients should not sit close to each other. Hand hygiene and social distancing should be underlined. If possible, patients should wear a mask in the service.
- Patients should not be in close contact in the waiting rooms before the dialysis sessions. Much more attention should be paid to hygiene rules.
- There must be a distance of at least 2 meters between patient beds in the dialysis center.
- Patients should not eat during the dialysis sessions.
- Patients and employees must wear a surgical mask to prevent asymptomatic transmission.
- All staff working on dialysis should be monitored for fever and symptoms daily. In any suspicious condition, they should be directed to detailed evaluation.
- It is essential to create isolated units in the outpatient centers to be prepared for COVID-19 infected patients as the disease spreads.
- Hepatitis B isolation rooms may be an option if there is no separate area in the dialysis facility unless there are hepatitis B surface antigen-positive patients.
- Frequency of dialysis sessions should not be shortened or paused not to effect dialysis quality.

### Question 5: If HD patients have a fever and/or dyspnea, how should we approach?

**Figure 1.**
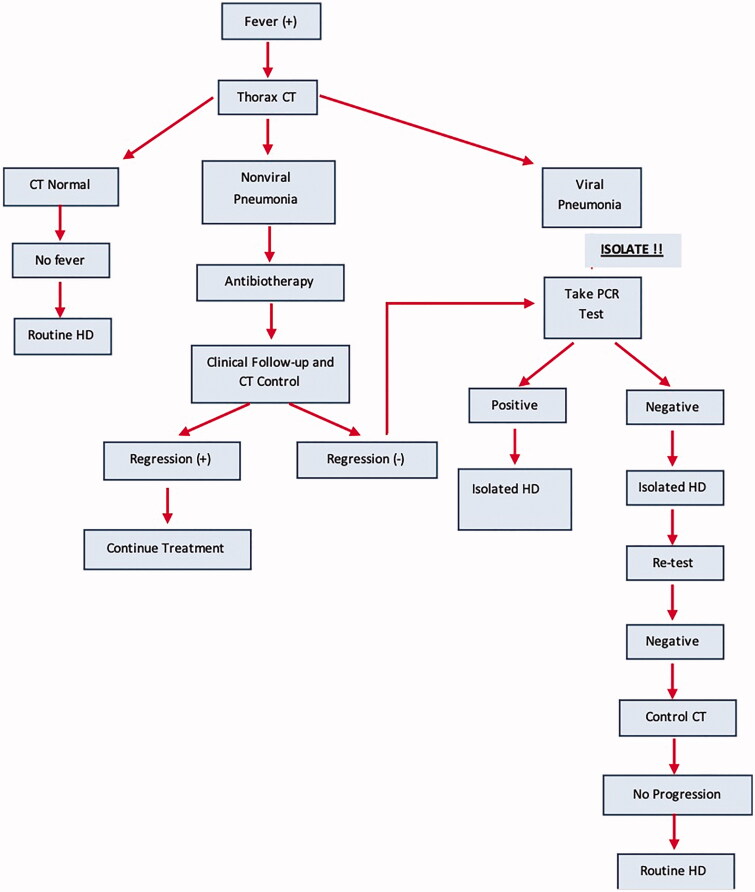
Management algorithm for HD patients with fever and/or dyspnea. CT: computerized tomography; HD: hemodialysis.

Answer: Actually, fever and/or shortness of breath are symptoms that are frequently encountered in dialysis patients, and in such cases, necessary physical and laboratory examinations are regularly performed. However, during the pandemic period first approach to these patients should be considering COVID-19. An algorithm approach from Wuhan can be useful in this regard (Figure 1) [38].

### Question 6: How should dialysis sessions be arranged for the COVID-19 infected HD patients during hospitalization?

Answer: COVID-19 infected HD patients should never be dialyzed in the same room with uninfected HD patients. If possible, portable dialysis machines may be used to keep COVID-19 patients in their rooms [19]. If there is no portable dialysis option, the patient should be dialyzed in an isolated room or area in the dialysis unit. These patients should be dialyzed at the end of the day. The room should be ventilated, and the dialysis machine should be properly disinfected after the session. If the patient is being followed up in the ICU; intermittent (IHD) or continuous renal replacement therapy (CRRT) can be performed at the bedside. Especially for patients in the ICU unit, performing CRRT instead of IHD may be preferred due to its beneficial effects on cytokine storm and inflammation, although there is no clear evidence. Medical staff performing dialysis should be experienced [19]. If it is possible the same dialysis staff should manage COVID-19 patients to decrease contact with uninfected patients. They should be kept in a continuous education program for the use of personal protective equipment during these procedures [33].

### Question 7: How outpatient dialysis centers should manage confirmed or suspected COVID-19 patients?

Answer: Outpatient clinics should direct suspected COVID-19 patient to hospital for a multidisciplinary evaluation. If the patient does not require hospitalization but confirmed, the patient should be dialyzed at the last session. A specific room should be reserved to prevent contamination. If it is not available, the patient should be dialyzed at a corner of the unit as far as possible from other patients.

### Question 8: Does HD patients need vitamin and mineral supplementation to strengthen their immune systems during this period?

Answer: Recent studies showed that exaggerated inflammation and cytokine storm were underlying causes related to high mortality in the course of COVID-19. Nowadays, treatment modalities that balance the immune system of such vulnerable patient groups to protect from the disease are essential. Several vitamins (namely vitamins C, D, E), trace elements like zinc, and probiotics have been proposed as a booster of the immune system to protect and combat infectious conditions [39]. These vitamins and elements are generally insufficient in hemodialysis patients. Although existing data is inadequate, supplementation with multiple micronutrients and probiotics for their immune-boosting role may be tried. Such supplementations may modulate immune function and reduce the risk of COVID-19 and its severe course [39]. Better-designed human studies are necessary, especially in HD patients.

### Question 9: Is dose adjustment for antiviral treatment necessary for HD or PD patients?

Answer: There is no clear evidence about treatment options of COVID-19. Beyond that, there is not enough information about renal clearance of antiviral drugs used. At the moment, there are no specific dose adjustment recommendations for HD or PD patients. Detailed information can be found in the Table 2 presented in the AKI section above.

### Question 10: As the pandemic spreads through dialysis facilities, what precautions should be taken for uninfected HD patients?

Answer: It is evident that outpatient dialysis units are under significant risk of viral transmission. As the infected HD patient number increased, there would be challenges to protect uninfected HD patients and healthcare workers in the units. PD and home HD options are advantageous in providing patients’ isolation in this aspect. In this period, medical policies should be revisited to encourage nephrologists and patients for home dialysis options. Suitable HD patients might be switched to PD if the COVID-19 spread upsurge. After the pandemic, health policies should also be reevaluated to be compelling for home dialysis facilities.

## Covid 19 and kidney transplantation

### Question 1: Are kidney transplant patients at high risk?

Answer: The general understanding of COVID-19 is advancing, but still little is known about transplant recipients. Especially their clinical presentation, disease course, or outcomes remain limited. Transplant recipients appear to be at high risk for critical COVID-19 due to their immunosuppressed status and coexisting conditions like diabetes, hypertension, or heart disease. The duration of transplantation is also crucial for risk, since newly transplanted patients are under higher risk compared to long-term stable transplant patients. In the first 6–12 months of transplantation, patients are prone to infections because of high dose immunosuppressive treatment. Infection progression is straightforward, and clinical presentations are atypical in this period. Clinical data from USA emphasized that transplantation time is vital in transplant recipients with COVID-19. In a case series of 28 kidney transplant recipients, 50% of patients with COVID-19 were in the first five year of transplantation and 71% of these patients were in the first year [40]. At this time, the risk factors, presentations, and outcomes for COVID-19 infection have not been fully characterized in the transplantation setting. All transplant recipients should be evaluated according to their transplantation duration, drug dosage, and accompanying diseases individually. Since the diagnostic PCR test has high false negativity, a CT scan of the chest (low dose without contrast) should be preferred in case of any clinical suspicion.

### Question 2: What is the outcome of renal transplant patients with COVID-19?

Answer: Case reports of kidney transplant recipients from USA, UK, Spain, Wuhan, and Italy revealed that ages and transplantation time of patients might differ as 28–72 years and eight months to 12 years, respectively. Patients presented most often with a fever and/or cough, but atypical presentations with gastrointestinal problems like nausea, vomiting, and diarrhea were not rare. In all series, immunosuppressive drugs were discontinued, and in most of the cases, corticosteroid dose was increased. Patients were followed with antiviral therapy at the hospital. In two series from USA, it was mentioned that 22–34% of patients were followed at home with telemedicine. When all case series were evaluated, 65–72% of patients were followed in the hospital. 40–60% of these patients were clinically stable that they did not need ICU admission. Overall AKI and RRT incidence were 21–38% of all transplant patients. Patients’ mortality rate differs between 5 and 64% from center to center and according to different treatment modalities. Patients who received ATG (which decreases all T-cell subsets for many weeks) or any rejection treatment during the last 5-8 weeks had worse outcomes, even mortality. These patients were with the lowest CD3, CD4, and CD8 cell counts. As a result, kidney transplant recipients with COVID-19 are likely to have a critical disease course. Age, comorbidities, IS dose, during the first six months of transplantation, recently received any rejection treatment or ATG, make transplant patients prone to AKI, ICU admission, or mechanical ventilation. All patients should be evaluated individually and followed skeptically in any change of clinical condition [41–49].

### Question 3: Should cadaveric or living-donor kidney transplantation be delayed during this period?

Answer: Risks of COVID-19 donor-derived infections and/or risk of exposure to COVID-19 of the recipient after transplantation are not clearly known. According to the American Society of Transplantation, The Transplantation Society and ERA-EDTA recommendations, in countries with COVID-19 pandemics, temporary suspension of the living or deceased donor program should be considered. In cases, if the transplant is vital especially for deceased donor transplants, the donor should be carefully evaluated. Testing of upper and lower airway specimens by PCR/NAT of donors with concern for COVID-19 should be considered. Likewise, donors with unexplained respiratory failure leading to death should be excluded. In a case series of cadaveric kidney transplantation, 15% of patients were SARS-CoV-2 RNA test positive in donor. If possible, it would be better if transplant can be performed without induction therapy. In cases where induction therapy is absolutely necessary, IL 2 receptor antagonists may be preferred, and ATG use may be avoided [45,50–52]. Besides, it should be kept in mind that during the outbreak, medical staff and health-care capacity might be inadequate if massive widespread occurs, and this will adversely affect the quality of patient care after transplantation.

### Question 4: What shall we suggest to kidney transplant recipients during COVID-19?

Answer: Transplant recipients should be warned as; they appear to be at particularly high risk for critical COVID-19 illness due to chronic immunosuppression and coexisting conditions and they have to take care of all recommended procedures to avoid COVID-19 transmission. Notably, patients who are in the first year of transplantation should be much more cautious about prevention procedures. It is recommended to postpone the appointments in stable patients. Telemedicine applications which is discussed in Section F may be useful in these patients. Patients should be educated about the infectious symptoms and any atypical presentation, not to delay the diagnosis [52,53].

### Question 5: Should immunosuppressive dose be reduced in non-infected kidney transplant patients during the COVID-19 pandemic?

Answer: The transplantation team must be doubtful about decreasing immunosuppressive drug doses and decisions should be made on a case-by-case basis. The transplantation history of patients affects the expected rejection risks. Patients in first six months of transplantation are under higher risk in this sense. In a small number of studies, the risk of infection with COVID-19 in kidney transplant patients was similar to the general population, but with a more severe clinical presentation. Interestingly, the authors argued that immunosuppressive drugs in this setting could reduce the viral load. In recent data, SARS-CoV-2 replication was linked to active immunophile pathways, and tacrolimus strongly inhibited the growth of human coronaviruses like SARS-CoV, HCoV-NL63, and HCoV-229E in cell culture. Cyclosporin A (CsA) has also been identified as a potent replication inhibitor of various human or animal related CoVs [54]. Carbajo-Lozoya et al. demonstrated that CsA and FK506 derivatives inhibits HCoV-NL63 replication regardless of immunsupresstion [55]. These agents have anti-viral effect in low dose via peptidyl-prolyl isomerase and immunosuppressive effect in high dose via cellular phosphatase calcineurin. It is clear that; uncontrolled inflammation and cytokine storm play an essential role in mortality related to COVID-19. New treatment options are focused on reducing this uncontrolled immune response (IL-6, TNF alpha inhibitors, etc.). In this regard, both viral replication-reducing effects of immunosuppressive drugs, and their suppressive effects on exaggerated immune response, should also be considered [53]. As a result, unless patients have COVID-19 infection there is no need to reduce immunosuppressive drug dose, especially for not facing with rejection problems in this period.

### Question 6: If the kidney transplant recipient has COVID-19, what changes should be made in immunosuppressive treatment?

Answer: In the course of diagnosis and management of COVID-19, all transplant recipients should be reviewed on a case-by-case basis. The ideal treatment for kidney transplant recipients with COVID-19 remains uncertain at present. It is likely to reduce immunosuppression. Suggestions can be listed as follows:

- If mycophenolate or azathioprine is withheld and tacrolimus or cyclosporine decreased, higher dose systemic corticosteroids (40 mg/day İV) should be the option. Corticosteroids may provide immunomodulatory and anti-inflammatory benefits while protecting the graft [56].
- Patients with normal imaging and no or mild symptoms should be closely monitored in the hospital in case of a strong suspicion for COVID-19. Reducing immunosuppressive dose should be kept in mind according to the patient’s clinical course. All other infection-related causes should be ruled out carefully.
- Patients confirmed positive for COVID-19 with normal CT imaging should be hospitalized. Antiviral treatment should be started. Although firm recommendations are not possible in this case, mycophenolate or azathioprine have to be stopped. Calcineurin inhibitors (CNIs) and corticosteroids dosage should be reviewed in a daily manner. It has been suggested that acute inflammatory processes and progressive infection can be prevented in these patients, with the continuation of low-dose CNIs [53]. Complete discontinuation of CNIs is considered in any case of symptom progression, imaging abnormality, or drug-drug interaction with antiviral management.
- Patients confirmed positive for COVID-19 with imaging abnormality should be hospitalized and carefully followed in regard to severe infection (requiring intubation and ventilation). The most common recommendation is to continue corticosteroids 40 mg/day and discontinue mycophenolate or azathioprine. CNIs dose should be reduced, but discontinuation has not yet been clarified if the patient is not critically ill. Target blood through level for tacrolimus should be not more than 4–6 ng/ml [57].
- There is not enough evidence base for antiviral drug use (regarding pharmacokinetics and toxicity) in the transplant setting. Prolongation of the QT interval and alterations in the metabolism of tacrolimus should be kept in mind. Antivirals, including remdesivir, chloroquine, ribavirin, lopinavir-ritonavir, tocilizumab, and favipiravir are candidate drugs at present. But their efficacy and safety in the treatment of transplant recipients with moderate or severe COVID-19 are still in doubt. Since drug interactions are frequent, CNI blood through levels should be checked more frequently during the antiviral treatment, as protease inhibitors (lopinavir and ritonavir) can dramatically increase tacrolimus serum levels [57].
- In the course of COVID-19 infection, the negative SARS-CoV-2 RNA test should be confirmed twice, and patients should be socially isolated for 14 days after discharge. Regarding the management of immunosuppressive, each patient should be evaluated individually. Lopez et al. reported that tacrolimus might be restarted in half of its former dose, when the PCR test is negative (2 times), although radiological findings still persist. Two weeks after clinical and radiological recovery, the previous dose of tacrolimus and mycophenolate should be restarted stepwise. Currently, ongoing clinical trials are expected, where we can find more definite answers [44,57,58].

### Question 7: how should we treat graft rejection in case of COVID-19 (+) or COVID-19 (-) patients in this period?

Answer: Any data about transplant recipients with biopsy-proven acute rejection have not been published yet. In a case series of 5 kidney transplant patients with COVID-19, one of the patients followed as acute rejection because of increased serum creatinine and decreased urine output. The patient’s absolute leukocyte and neutrophil counts increased, and newly onset proteinuria was determined. The patient was given three days of methylprednisolone pulse therapy (500, 250, and 250 mg). Kidney biopsy findings were not presented, but plasma BUN, creatinine, and urine output returned to normal levels on the third day of treatment. During follow-up, the patient had fever and typical imaging findings for COVID-19 on a CT scan. After two weeks of treatment for pneumonia, the patient was discharged. As a recommendation, during an acute rejection episode in a transplant recipient with COVID-19, antiviral and supportive treatment should be the first option rather than the rejection management. If patient does not have COVID-19, 250 mg IV corticosteroid followed by 1 mg/kg 3 days oral dose and tocilizumab should be used in cellular type acute rejection. In case of humoral rejection, IVIG, plasmapheresis, and tocilizumab are recommended treatment options, which are already used in the course of COVID-19 [41,58–60].

### Question 8: What are the COVID-19 related problems in the graft?

Answer: The frequency of COVID-19 related AKI is not known at this time in kidney transplant patients. Recently, 6 cases of transplant recipients with COVID-19 have been reported. The authors said that after antiviral treatment and 40 mg/day IV corticosteroid, BUN and serum creatinine levels turned baseline levels in all patients. However, renal biopsy findings of these patients have not been reported. Immunosuppressive drugs may play a serious role in preventing kidney damage in case of COVID-19 with their immunomodulatory and anti-inflammatory properties [41,58].

In conclusion, although immunosuppressed individuals have an increased risk of severe COVID-19, kidney transplant recipients have to be evaluated in two aspects. First, IS drugs used in the transplantation setting might be useful during COVID-19 course concerning immunomodulation effect and control of cytokine storm. Second, transplant recipients are well-informed about infectious diseases and their protection ways. During the pandemic, patients should be followed with telemedicine, and all patients should be informed about the atypical course of infection.

## COVID-19 and chronic kidney disease (CKD) with glomerular disease

### Question 1: Do primary or secondary glomerulonephritis and CKD pose a risk for the COVID-19?

Answer: There is limited data about risk of patients with primary and secondary glomerulonephritis (GN) and CKD during the COVID-19 pandemic. However, these patients already have tendency for various infections. Underlying glomerular disease-related adaptive and innate immune system problems, IS drugs used in the treatment of GN, and immunosuppressive state related to uremia itself are major mechanisms for increased risk of infection [61,62]. All CKD patients therefore needs a regular immunization program for several infectious agents. Taken together, it is likely that this group will have a more severe course when they are infected. On the other hand, it was shown that patients using corticosteroids and having COVID-19 pneumonia had milder symptoms without any respiratory distress [63]. There is not any publication about GN and COVID-19 relations at this time. But this group of patients should be alert about the preventive measures.

### Question 2: Is it necessary to modify CKD management in case of COVID-19?

Answer: Anemia and metabolic bone disease management are corner stone in the control of morbidity and mortality in CKD. There is no currently available data about patients with CKD and COVID-19 but also no supportive suggestion to stop these treatments. Calcitriol and vitamin D analogs are specially approved in the anti-inflammatory and renin-angiotensin-aldosterone blocker effects beyond their mineral-bone disease control [64]. Many studies are showing vitamin D treatment is also related to decreased mortality with the help of proteinuria control [65]. It is reasonable to say that vitamin D may control the infection course with its anti-inflammatory effects and also may protect cells from virus entrance with the RAS blocker effect. Studies with vitamin D supplementation in coronavirus infected patients are necessary to understand real outcomes. Maintenance IV iron treatment should be avoided during the active systemic infection as routinely done. Recommended vaccines for CKD patients should not be postponed during the pandemic. Patients with malnutrition should be evaluated particularly since they are under increased risk for infections further.

### Question 3: Is it necessary to stop RAAS inhibitors in GN and CKD patients during the COVID-19 pandemic?

Answer: Very little is known about treatment options of primary GN and CKD during the COVID-19 outbreak. RAAS inhibitors (angiotensin-converting enzyme (ACE) inhibitors or angiotensin receptor blockers (ARB)) are used for their antiproteinuric effect in glomerular diseases. The effects of RAS inhibitors on ACE-2 receptors in COVID-19 patients have been previously discussed in the ‘Hypertension’ section of this article. In summary, based on current evidence, RAAS blockers should be continued in patients with GN and CKD due to its protective effects on cardiovascular complications, proteinuria, and glomerular damage.

### Question 4: What do we know about diabetic nephropathy and COVID-19?

Answer: People with diabetes have been identified as being at increased risk of serious illness from COVID-19. Although data is not in high-quality, Wu et al. reported that 7.3% of patients who died were diabetic. In this study, 44,672 patients were evaluated [66]. The underlying immunosuppressive state in diabetes is generally related to hyperglycemia itself or circulatory and autonomic problems. Patients with Diabetic Nephropathy (DN) are reinforced for the risk of COVID-19 with diabetes and CKD concomitantly. Patients with DN should be under intensive glycemic control during this period, and vaccination for influenza and pneumococcal diseases should be scheduled [67]. During the infection course, SGLT2i should be revised carefully in DN patients. Oral antidiabetic drugs (OAD) and SGLT2i should be withheld in severe cases due to their GFR and nutritional status. Insulin regimens should be the first option in such cases [68].

### Question 5: Is it necessary to modify immunosuppressive drugs in GN patients to decrease the risk of COVID-19 infection?

Answer: Patients with GN who are on IS treatment should be evaluated case-by-case. Although the literature is continually being updated, according to our current knowledge, we do not recommend stopping IS treatments in stable GN patients. Tapering of IS in stable patients should be as planned according to underlying disease. Hydroxy chloroquine phosphate (HCQ) use in GN (other than ongoing use like SLE) in prophylaxis is not recommended.

### Question 6: Is it necessary to modify immunosuppressive drugs in GN patients with COVID-19 infection?

Answer: In GN patients with confirmed COVID-infection but no or mild symptoms, high dose maintenance treatments (pulse steroid, cyclophosphamide, rituximab, etc.) should be postponed. The patients who had rituximab in the last 12 months should be evaluated for hypogammaglobulinemia (serum IgG level < 500 mg/dL) and intravenous immunoglobulin (200–300 mg/kg every 3–4 weeks) should be recommended in case of hypogammaglobulinemia. Corticosteroids should not be stopped. Other IS treatments (antimetabolites, CNIs, etc.) should be discussed case-by-case and reorganized as in kidney transplant recipients [69]. If the patient has severe COVID-19 infection; treatment options should be evaluated in a multidisciplinary approach. Nephrologists, infection disease specialist, and intensive care specialists should decide together to IS drug maintenance and antiviral options. Antiviral treatment type and dose should be carefully revised according to GFR changes during the infection course. Corticosteroids may be beneficial due to their cytokine storm-reducing effects in these patients. But corticosteroids dose is not precise at this time. This issue was discussed in detail in the transplantation section. Discontinuation of corticosteroids abruptly may increase the risk of adrenal insufficiency. The rapid progression of GN or relapses in vasculitic diseases should be taken into consideration.

### Question 7: Should we perform kidney biopsy during the COVID-19 pandemic?

Answer: Patients should be evaluated individually in a risk-benefit way, and biopsy should be performed in urgent cases. For example, in case of nephrotic syndrome and anti-PLA2R (+), patient should be accepted as primary membranous nephropathy, and immunosuppressive treatment should be deferred according to patient condition. In case of life-threatening situations, biopsy and following IS treatment should be scheduled as soon as possible.

### Question 8: Should newly diagnosed primary GN patients receive is treatment during the COVID-19 period?

Answer: Patients newly diagnosed with primary GN without COVID-19 infection should be evaluated according to biopsy findings and kidney function tests. If it is thought to be a slowly progressive disease, patient should be followed with conservative treatment options rather than ISs. In case of newly diagnosed primary GN patients with moderate to severe COVID-19 infection, ISs should be postponed. RAAS blockade, low molecular weight heparin, diuretic, and statin therapy will be more suitable in this period. However, in patients with life-threatening glomerular diseases (ANCA-associated vasculitis with lung involvement, lupus nephritis with other organ involvement, etc.), plasmapheresis can be the first option in addition to corticosteroids (IV mini-pulse-250mgr and 1 mg/kg/day in the following days). Such patients should be reevaluated daily with respect to drug adjustments, to be sure with risks and benefits.

### Question 9: Is rapidly progressive GN seen in related to COVID-19 infection? How do we manage a patient with a newly diagnosed Pauci immune glomerulonephritis during COVID1-9 infection?

Answer: Based on current evidence, we are not sure. In a series of 701 patients, proteinuria and hematuria were present in 44 and 27% of patients, respectively. 13% of patients presented with GFR < 60 mL/min and 5% with AKI [13]. These presentations all suggested evidence of glomerular injury. In a recent case report, an African American patient with COVID-19 who had a rapid decline in kidney function was reported. Renal biopsy of the patient revealed a collapsing glomerulopathy. In any clinical condition of rapidly progressive GN (RPGN) in COVID-19 infected patients, patients should be evaluated individually in terms of benefits and life-treating side effects. A kidney biopsy is controversial, depends on patients’ clinical conditions. Corticosteroid treatment is skeptical and should be balanced with the worsening of infection control. Plasmapheresis/plasma exchange might be other option instead of high-dose corticosteroids. Again, in a patient with newly diagnosed pauci immune glomerulonephritis and COVID-19, in life-threatening conditions related to vasculitis (vasculitis with lung involvement, lupus nephritis with other organ involvement, etc.), IS treatment should be in mind. Although there is no evident data, IVIG might be used according to infection severity and vasculitis course.

### Question 10: Should routine IV cyclophosphamide treatment be continued in a patient who has recently been diagnosed with Pauci immune glomerulonephritis during the COVID-19 pandemic?

Answer: High-dose maintenance treatments (such as pulse steroids and cyclophosphamide) should be postponed, although there is no evidence this makes a difference. IV cyclophosphamide may be changed into oral form to prevent hospital admissions. Patients should be carefully followed in respect to relapses and vasculitis related life-threatening conditions. In patients that need maintenance treatments, COVID-19 screening should be optimally performed even with a CT scan or virus-PCR test if necessary.

### Question 11: Is there any drug interaction between is drugs and antiviral drugs used in COVID-19 infection?

Answer: Although most of the antiviral drugs used in COVID-19 are newly used ones, possible interactions with the IS drugs used in GN might be as following [32];

- Azathioprine interacts with chloroquine, hydroxychloroquine, ribavirin, and tocilizumab. Dose adjustment and close monitoring may be required.
- Atazanavir, lopinavir/ritonavir, chloroquine, hydroxychloroquine increases the levels of cyclosporine and tacrolimus. Dose adjustment and close monitoring may be required for CNIs. Tocilizumab also weakly reduces the effect of cyclosporine.
- Mycophenolate potentially interacts with lopinavir/ritonavir. Dose adjustment and close monitoring may be required.

## COVID-19 and telemedicine

### Question 1: Are remote access (telemedicine) applications helpful for renal patients during the COVID-19 pandemics?

Answer: During the pandemic, strict lockdown rules may block access of patients for routine hospital appointments. Nowadays, technological improvements make it easier to reach patients at home through tele-medicine practices such as cell phones, smartphone applications, video calls, smartwatch applications. Elderly patients who may not efficiently use these technologies may be followed with phone calls by asking signs and symptoms and home measurements of blood pressure. Dose changes may still be done with telemedicine, but in case of drug changes or laboratory tests, patients should come to the hospital with appropriate precautions. Considering that most of our patients are within the risk group for the COVID-19, remote access methods will help patients in routine follow-up and hypertensive emergency conditions to guide for hospitalization.

## Conclusion

During the last four months, almost all practices were completely engaged with COVID-19 infections. Patients with hypertension, chronic kidney disease, maintenance dialysis treatments or kidney transplant are also a high-risk group for the COVID-19. Acute kidney injury due to infection course or direct viral injury should be another growing concern as the pandemic continues. Nephrologists should manage this high-risk population with relevant recommendations on an up to date manner. The COVID-19 literature was so prolific, but it was also very preliminary and sometimes contained no whit for evidence-base care of these patients. Nephrologists continue to practice while continuing to learn how to better practice for COVID-19 related kidney problems. We believe that every bit of information -whether regularly peer-reviewed or just a preprint – may help us for care. But the information should be carefully ‘stilled and disinfected with soapy water’ before inculcating for regular practice. This review tried to summarize the best available information. Nephrologists, however, continue to learn and gather more data and high-quality studies while fighting against COVID-19. We all hope our patients’ population and ourselves will get through this process with minimal or no damage.
